# Comparison of efficacy of three devices of manual positive pressure ventilation: a mannequin-based study

**DOI:** 10.1186/s13052-015-0131-9

**Published:** 2015-03-31

**Authors:** Somashekhar M Nimbalkar, Suman Rao PN, Saudamini V Nesargi, Ashish R Dongara, Swarnarekha Bhat

**Affiliations:** Department of Pediatrics, Pramukhswami Medical College, Karamsad, Anand, Gujarat 388325 India; Department of Neonatology, St. Johns’ Medical College, Bengaluru, Karnataka 560034 India; Department of Neonatology and Pediatrics Mazumdhar Shaw Medical Centre, Narayana Health City, Bengaluru, Karnataka 560034 India

**Keywords:** Neonatal resuscitation, T-piece resuscitator, Self-inflating bag, Flow-inflating bag

## Abstract

**Background:**

We compared the efficacy of and consistency in manual ventilation by trained healthcare professionals using three devices: self-inflating bag, flow-inflating bag, and T-piece resuscitator.

**Methods:**

Prospective analytical study at a level III Neonatal unit of a tertiary care hospital. Forty participants (consultants, postgraduates, interns, and neonatal nurses – 10 each) manually ventilated a mannequin with the above three devices for three minutes each. This procedure was video recorded. The pressure delivered during the three minutes and the breath rates for the first minute, second minute, and third minute were analyzed. Descriptive statistics were used to describe the study population and group statistics were used for various parameters of interest. Factorial analysis of variance was conducted to determine the main effects of device and specialty of users.

**Results:**

The mean (SD) peak inspiratory pressure of T-piece resuscitator was 16.5 (1.2), self-inflating bag (SIB) was 20.7 (4.4), and flow-inflating bag (AB) was 21.2 (5.0). The mean (SD) positive end expiratory pressure of T-piece resuscitator was 4.7 (0.9) cm of H_2_O and AB was 1.8 (1.7) cm of H_2_O. The maximum pressure delivered by T-piece resuscitator was 17.5, AB was 26.2, and SIB was 25.2 cm of H_2_O. Clinically appropriate breath rates were delivered using all of the devices. More effective breath rates were delivered using T-piece. There was no significant difference among the professional groups.

**Conclusions:**

The T-piece resuscitator provides the most consistent pressures and is most effective. Level of training has no influence on pressures delivered during manual ventilation.

## Background

The transition of a neonate from the intra-uterine environment to the extra-uterine environment at the time of birth is a major one. Almost 90% of the neonates are able to accomplish this transition without any external assistance. The remaining 10% require some form of support, of which 1% require extensive resuscitation including positive pressure ventilation [1]. Ventilation is the most important aspect of neonatal resuscitation [1]. In developing countries, effective ventilation strategies including positive pressure ventilation can reduce the early neonatal mortality rate by almost 45% and also reduce the incidence of fresh stillbirths [2,3].

The most commonly used devices for providing manual positive pressure ventilation are self-inflating bags (SIB), flow-inflating or anesthetic bags (AB), and T-piece resuscitators [4]. They are essential not only during neonatal resuscitation, but also for assessment and management during acute deterioration, equipment failure, and transport [5]. All three devices have their own advantages and shortcomings. Improper use of these devices can lead to barotrauma and volutrauma (because of high peak inspiratory pressures (PIP) and tidal volume (TV)), atelectotrauma (due to improper positive end expiratory pressure (PEEP)), and super oxygenation injuries to the lung and other organs (due to excess FiO_2_) [6,7]. The pressure delivered depends on method of delivery as well as the operators involved [8].

The equipment used to provide positive pressure ventilation to newborns needing resuscitation at delivery varies among institutions. In India, the method currently used in most neonatal units is SIB. The National Neonatal Resuscitation Program recommends use of a SIB, while the 2010 Neonatal Resuscitation Program guidelines recommend all three: the SIB, AB, and the T-piece resuscitator [1,9]. The aim of our study was to compare the effectiveness and consistency of manual ventilation by trained healthcare professionals using all three devices.

The primary objective was to compare the PIP, PEEP, mean airway pressure, and breath-rate delivered during manual positive pressure ventilation using the three devices available for neonatal resuscitation. The secondary objective was to determine whether level of training influenced positive pressure ventilation.

## Methods

### Design

We conducted a prospective analytical study at a level III neonatal intensive care unit (NICU) of a tertiary care hospital. Healthcare personnel involved in the care of neonates who had received training in neonatal resuscitation were included in the study. These included nurses, interns, postgraduates, and consultants.

### Sample size

To detect a difference in pressure of 2 cm of H_2_O among the resuscitation devices, a sample size of 27 subjects was required for a study with a power of 80% at a significance level of 0.05. We included 10 participants from each group to give a total of 40 subjects for each device.

### Equipment

A Laerdal neonatal mannequin with inflatable lungs was used. Adequate chest expansion (Peak Pressure > 15 cm of H_2_O) was indicated by a green light [10]. A 500 ml silicone SIB manufactured by Anaesthetics India Pvt. Ltd. (India) without a PEEP valve and with a pop off valve set at 30–35 cm of H_2_O, Fisher & Paykel (USA), infant T-piece resuscitator – 900 series also known as Neopuff™, and AB also known as Jackson Rees bag were used. A round shaped facemask with cushion was used with all the devices [11].

### Methods

The participants were asked to manually ventilate a mannequin. The participants were familiarized with the equipment and were allowed to test the equipment for a maximum period of five minutes prior to evaluation. During this time, they were asked to note the chest expansion, which was indicated by a green indicator light. The green light indicated administration of an effective breath and administering a peak pressure exceeding 15 cm of H_2_O. During evaluation, the participants were not allowed to visualize the green light. The participants were to determine the adequacy of ventilation based on chest rise only. The rate of oxygen flow was kept at 10 liters/minute for all the three devices. A PIP of 18 cm of H_2_O and PEEP of 5 was set for the T-piece resuscitator.

The study commenced with the subject manually ventilating the mannequin for three minutes first with the T-piece resuscitator, followed by SIB, followed by the AB. The pressures delivered during ventilation were measured on the same manometer for all three ventilation devices. The manometer was calibrated after use of each device. The subjects could not visualize the manometer or the green indicator during the entire study. This decision was made based on evidence that visualization of the manometer does not affect performance [12]. The pressure delivered during the three minutes intervals with each resuscitation device was video recorded by the principle investigator. Another observer documented the lighting of the green indicator.

The pressures delivered during the first 10 seconds of each minute were noted for each device by replaying the video i.e. 0–10 seconds, 61–70 seconds, and 121 to 130 seconds of each method. This was done for feasibility issues. Breath rate for the entire first minute, second minute, and third minute were noted. The effective breath rate was determined by manually counting the number of times the green indicator light was activated. The data were entered into a predesigned proforma. The study was approved by the St John’s Medical College & Hospital Institutional Ethics Committee, Ground Floor, St. John’s Medical College, Bangalore. Written informed consent was obtained by the participants.

Primary outcomes were determined by the PIP and PEEP difference among the devices. Secondary outcomes were determined by difference in the breath rates delivered through each device, maximum pressures delivered by each device, and difference in the parameters by training level of the personnel resuscitating.

### Statistical analysis

Descriptive statistics were used to describe the study population and group statistics for various parameters of interest. Factorial analysis of variance was conducted to determine main effects of device (T-piece resuscitator, SIB, and AB) and specialty of users (nurse, intern, resident, and consultant) and interactions between them (if any). SPSS 14.0 was used for analyzing the data.

## Results

### Study groups

The present study included a total 40 participants of whom 10 were consultants, 10 were postgraduate students, 10 were interns, and 10 were neonatal nurses. A total of 17,636 inflations were recorded from the participants using each of the three device combinations.

### Peak inspiratory pressure

The mean (SD) PIP was 19.5 (4.4) cm of H_2_O. The mean (SD) PIP of T-piece resuscitator was 16.5 (1.2) cm of H_2_O, SIB was 20.7 (4.4) cm of H_2_O, and AB was 21.2 (5.1) cm of H_2_O. There was no significant difference among the professional groups (p = 0.631). There was significant difference between T-piece resuscitator and SIB (p < 0.001) and AB (p < 0.001), but no difference between SIB and AB (p = 0.62) (Table 1). Post-hoc test for groups are not provided, as group effect was not significant.

**Table 1:** **Parameters delivered by use of various PPV devices**

**Professional qualification**	**Device**	**Mean PIP (cm of H**_**2**_ **O)(SD)**	**Mean PEEP (cm of H**_**2**_ **O)(SD)**	**Mean breath rate (BPM)(SD)**	**Effective breath rate (%)(SD)**
Consultants	T-piece	16.1(1.2)	4.3(1)	50.3(11.5)	93(10)
	AB	21.0(3.2)	0.8(0.7)	46.9(15)	79(26)
	SIB	21.1(3.4)		54.3(10)	90(24)
Residents	T-piece	16.3(1.2)	4.3(0.9)	42.5(10)	92(11)
	AB	22.1(7.3)	3.0(2.2)	35.5(13.8)	90(17)
	SIB	22.0(4.3)		46.3(20.9)	76(29)
Interns	T-piece	17.2(0.9)	5.2(0.4)	49.4(14.2)	93(11)
	AB	19.7(3.4)	1.6(1.5)	38.1(17.1)	81(25)
	SIB	21.5(3.8)		48(21.7)	78(22)
Nurses	T-piece	16.3(1.3)	4.7(0.9)	53.8(11.6)	77(23)
	AB	21.8(5.8)	1.9(1.5)	48.1(13.5)	74(26)
	SIB	18.3(5.6)		71.7(12.4)	52(42)
Overall	T-piece	16.5(1.2)	4.7(0.9)	49.0(12.2)	89(16)
	AB	21.2(5.1)	1.8(1.7)	42.2(15.4)	81(24)
	SIB	20.7(4.4)		55.1(19.3)	74(32)

### Positive end expiratory pressure

The mean (SD) PEEP of T-piece resuscitator was 4.7 (0.9) cm of H_2_O and AB was 1.8 (1.7) cm of H_2_O. There was a significant difference among the professional groups (p = 0.033). There was significant difference between T-piece resuscitator and AB (p < 0.001) (Table 1). Post-hoc test for ventilation devices are not provided as PEEP is not provided by SIB.

### Maximum pressures

The maximum pressure delivered by the T-piece resuscitator was 17.5 cm of H_2_O, AB was 26.7 cm of H_2_O and SIB was 25.7 cm of H_2_O. There was no significant difference among the professional groups (p = 0.818). There was significant difference between the maximum pressures delivered by T-piece resuscitator and AB (p < 0.001) and SIB (p < 0.001), but no difference in between the maximum pressures of SIB and AB (p = 0.331).

### Ventilation rates

The mean (SD) rate delivered by T-piece resuscitator was 49.0 (12.2) breaths per minute (bpm), AB was 42.2 (15.4) bpm, and SIB was 55.1 (19.3) bpm. There was significant difference in breath rates among the professional groups (p < 0.001) (Table 1). The breath rates using AB were significantly less than those of the T-piece resuscitator (p = 0.041) and SIB (p < 0.001), but this difference was not significant clinically.

### Effective breath rates

The percentage of effective breaths delivered using T-piece resuscitator, AB, and SIB were 89 (16) %, 81 (24) % and 74 (32) %, respectively. There was significant difference in effective breath rate among the professional groups (p < 0.001) (Table 1). There was significant difference in the effective breaths delivered using different devices (p = 0.022). There was no relationship between the operator groups and devices. T-piece resuscitator delivered breaths more effectively than SIB (p = 0.006), but equivalent to AB (p = 0.139). AB was equivalent to SIB (p = 0.187). Nurses delivered a significantly lower number of effective breaths than consultants (p = 0.002), residents (p = 0.004), and interns (p = 0.009) (Table 1). No differences were found among the other groups.

All the residents, nurses, and consultants reported that they are comfortable with resuscitation and had been involved in resuscitation in the past one year. Interns were not involved during the past year and they were not comfortable with resuscitation. The most preferred device for ventilation was the T-piece resuscitator (72.5%).

## Discussion

The present study shows that there is a statistically significant difference between the T-piece resuscitator and the AB bag as well as the SIB in mean PIP, mean PEEP, maximum pressure delivered and percentage of effective breaths. Hussey et al. found the AB and the T-piece resuscitator similar in mean PIP and mean airway pressures delivered if compared to the SIB. This difference also would be clinically significant. The T-piece resuscitator was superior in delivering consistent and effective ventilation. Moreover, the T-piece resuscitator protected against barotraumas as evidenced by the lower maximum pressure delivered than the other devices. This consistency is an inherent feature of the T-piece resuscitator as PIP and PEEP are set previously to the procedure by the operator, and thereby are not dependent on operator skill. The maximum pressure delivered by the T-piece resuscitator was 17.5 cm of H_2_O, AB was 26.3 cm of H_2_O, and SIB was 25.3 cm of H_2_O. The mean PIP of T-piece resuscitator was 16.5 cm of H_2_O, which was less than the set target value of 18. Mean PIP of a SIB and AB was higher than the expected PIP. This pattern is also observed in other studies [13,14]. The PEEP was delivered reliably only using the T-piece resuscitator. PEEP delivered by AB was inconsistent and at times even reached 12 cm of H_2_O, which is out of the limits reported by the current guidelines. PEEP was not delivered by the SIB. We didn’t use a SIB with PEEP valve, as it is not routinely available and utilized in India. This finding of inconsistent PEEP delivery with AB was not found in other studies. Conversely, other studies by Hussey et al., O’Donnell et al., and Roegholt et al., evidenced that the T-piece resuscitator proved to be better and consistent in effectively delivering PEEP [6,8,15,16].

The present study differs from those by Finer et al., Hussey et al., and O’Donnell et al. in that the participants of those studies had access to manometers while bagging the mannequin, which would allow them to tailor the pressures delivered [3,4,7]. Zmora and Merritt demonstrated that target ventilatory pressure were achieved 72% of the time while using a manometer, compared to only 18% while, not using it [17]. There is also some evidence that using a manometer does not affect the effectiveness of manual ventilation [6]. Most neonatal resuscitation teams in our scenarios will not have a manometer and would depend on the clinical signs to judge the adequacy of ventilation. Our participants gave manual ventilation pressures based on chest rise and had no knowledge of the manometer pressures.

Surprisingly, the groups used the AB as effectively as the SIB, even though this bag was not routinely used by the participants. The SIB delivered the lowest number of effective breaths (73.9%). The T-piece resuscitator delivered the maximum number of effective ventilations (88.9%). This difference was observed as T-piece and AB can be utilized only if the seal is adequate. Finer et al. compared different groups based on level of training for the three devices and detected a difference among groups for the various devices [8]. Other studies do not detect a difference among the groups for any of the devices [6,7,13]. In the present study, no correlation was found between qualification of the operator and pressures achieved except PEEP while providing manual positive pressure ventilation, but breath-rate per minute and effective breath-rate varied with qualification. This is probably due to the fact that consultants, and residents routinely performed neonatal resuscitation, and thereby were able to perform better than nurses. Twenty-nine (72.5%) of the participants, preferred the T-piece resuscitator, while four participants preferred the AB and seven participants preferred the SIB.

The main limitation of the present study is that it was done on a mannequin and the scenario in the delivery/operation room differs greatly. We utilized a low fidelity mannequin and this adds to the potential for bias. In a resource-limited nation like India, availability of pressurized gas source can be an issue. Therefore the SIB can prove to be more feasible during neonatal resuscitation. There are some recent studies that state that in spite of showing better results with mannequins, the T-piece resuscitator didn’t show improved neonatal outcomes [14,15]. Another drawback was the lack of randomization of the order in which the devices were used. This could introduce the potential for fatigue and practice effect bias. Further research is required to provide crisper recommendations.

## Conclusion

Among the three devices of manual positive pressure ventilation recommended for neonatal resuscitation, the most consistent and effective pressure was delivered by the T-piece resuscitator. Qualification of the operator did not have an influence on the pressures delivered during positive pressure ventilation.

## Abbreviations

SIB: Self inflating bag
AB: Anaesthetics bag
PIP: Peak inspiratory pressure
TV: Tidal volume
PEEP: Peak end expiratory pressure
SD: Standard deviation
